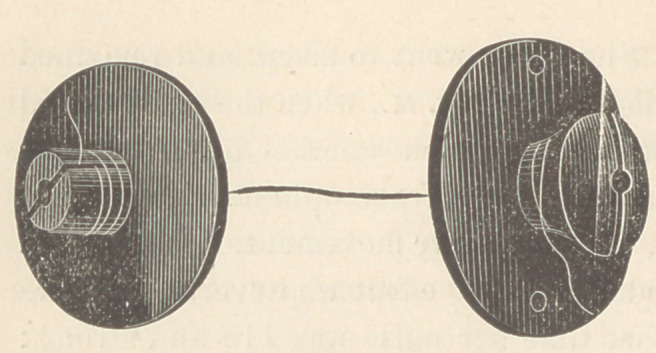# Dr. H. H. Clark’s New Suture Button

**Published:** 1882-07

**Authors:** 


### ARTICLE V.

### DR. H. H. CLARK'S NEW SUTURE BUTTON.

The small number of appliances adapted to a great many operations, together with the wide range of utility and easy application of this button, is my excuse for calling the attention of the profession to that which supplies a long felt, yet so far unsupplied want, to wit, a simple yet reliable suture, entirely under the control of the surgeon at any and all stages of the treatment of a wound regardless of conditions. Made of vulcanite, highly polished, they are easily cleaned, antiseptic, light, compact and so strong that they are practically indestructible.

The accompanying cut represents but one of a number of forms, but embodies the principle common to all, the securing of the ends of the suture. As will be readily seen, it is a flat disk

(in other forms the disk is oval of any desired length and width) with an upright stem or shank, with a central round slot passing from center of bottom of disk to top of the stem, which is also saw slotted down to the top of the disk.

The round disks are made in five different sizes. Flax, silk or wire can be used for suture, but I prefer wire especially in large wounds.

There is also a slot at each edge of the disk opposite each end of the saw slot. These are intended for the passage of sutures, the ends of which are secured in the saw slot. The disks being one inch across, three sutures can be used with one button leaving the sutures half an inch apart, by using an oval button or a number of small round ones, any desired number of sutures can be obtained. The sutures can be entered as far away from the edges of the wound, and passed as deeply into the tissues as may be deemed necessary to secure a firm and continuous hold for an unlimited period of time. A single line of buttons immediately over the wound or a row at each side and then laced across will secure the most perfect coaptation. When placed directly over the wound, the flat surface of the button causes a very perfect approximation of integument, securing as small and as perfect a cicatrix as it is possible for art to obtain. In using them on each side, pressure may be applied and while doing so a collodion coating given immediately over the wound then cross-binders applied, and over all a simple antiseptic gauze. By this means perfect inspection of the wound is obtained with very little trouble, and the parts are so secure that they may be freely handled without danger of disturbing the most delicate adhesions.

Their use as tractors to draw together muscles divided in their transverse is readily apparent. Every practical surgeon has seen the swelling so great that the sutures have rapidly cut or ulcerated their way out, or he has been obliged to cut them in order to relieve the great tension of the parts, thereby losing their benefit at the moment when most needed, namely, when the inflammation subsides. His adhesive strap failing to give requisite support the newly formed delicate adhesions give way leaving a gaping wound to fill and close by granulation. All this can be avoided by using sufficient length of suture and wrapping the surplus around the stems, and using as may be indicated either slacking or tightening the suture. In amputations, by starting it one to two inches on the open surface and bringing out through the integument at from three to four inches from the cut edge of the integument, upon closing the sutures the muscular structure will be forced or rolled in over the end of the bone, closing every part so strongly that very little of any cavity is left. The sutures can be put in and the wound left open any desired time and so permit all oozing to cease and the surface to glaze and so lessen the chances of the accumulation of fluids into any unavoidable pocket. The benefits from adhesive plaster may be had by using strips with button holes in it as tractors, to connect the rows of buttons, slipping the holes over their shanks.

In case of formation of pus or the occurrence of secondary hemorrhage requiring opening up the stump, they can be loosened up, the wound washed out, or, if need be, drawn out of the way and after accomplishing the purpose of the surgeon again closed.

In cases attended with sloughing of the lips of the wound, they will, if passed in deeply and broad of the wound, still keep their hold and so save the approximation and hasten repair. In ovariotomy operations on the female perineum and in plastic surgery generally, a wide field for their use presents. I respectfully submit them to the judgment of the many honest men composing the ranks of medicine and surgery, and dedicate them to their use for the benefit of suffering humanity.

## Figures and Tables

**Figure f1:**